# Supplementary material for: Regulatory remodeling in the allo-tetraploid frog Xenopus laevis
Source: Genome Biol. 2017 Oct 24;18:198. doi: 10.1186/s13059-017-1335-7 (PMC5655803; doi:10.1186/s13059-017-1335-7)
Supplement: Supplementary file 3 — Containing supplemental figures. (DOCX 1833 kb) [file 13059_2017_1335_MOESM3_ESM.docx]

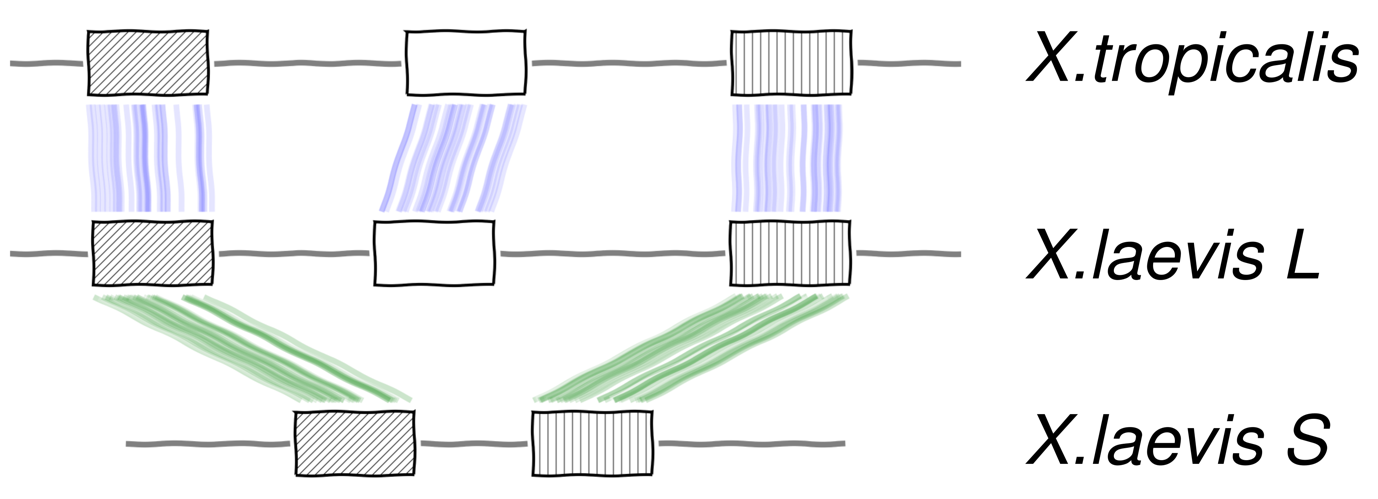


Figure S1. Strategy for calling deletions based on blocks of sequence conserved between one *X. laevis* subgenome and *X. tropicalis*, but lost from the other subgenome.


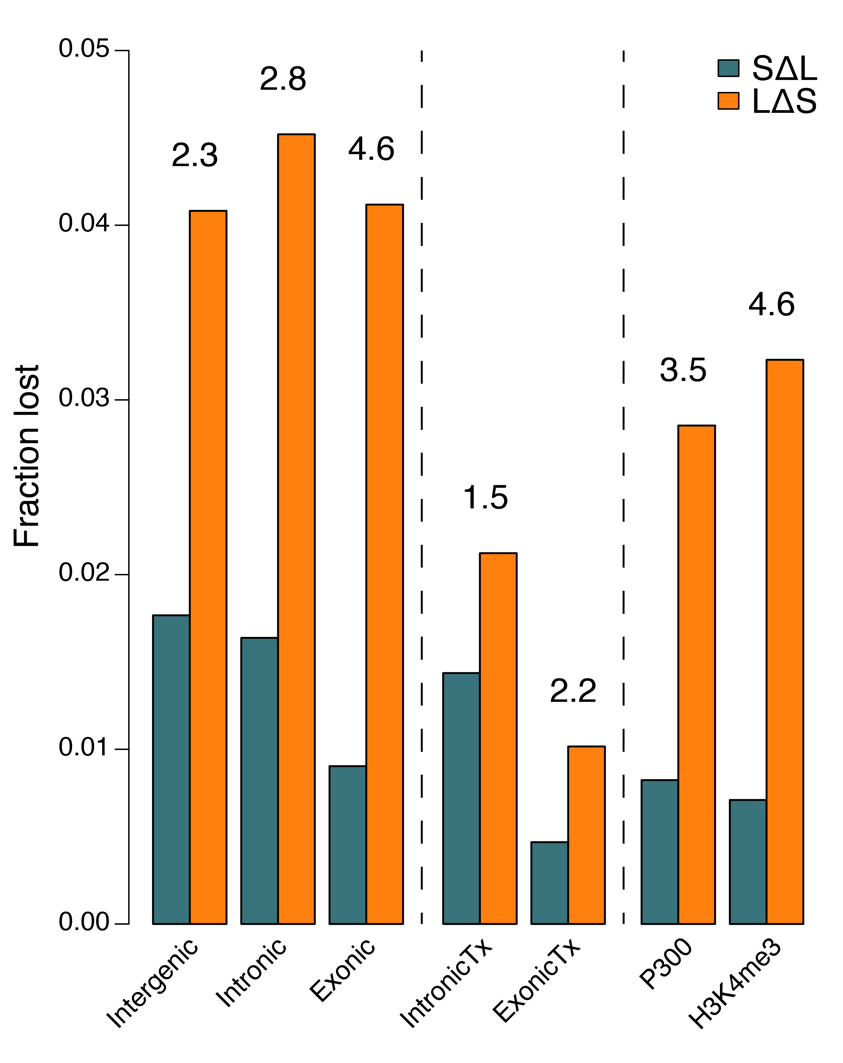


Figure S2: Fraction of genomic regions lost by deletions. Numbers on top of the bars represent the ratio of the fraction lost in S relative to the one lost in L. Intergenic: 1kb distance from a gene. Intronic: introns. Exonic: UTRs + CDS. IntronicTx: introns from genes actively transcribed. ExonicTx: Exons from genes actively transcribed. p300: genomic fragments having a p300 peak. H3K4me3: genomic fragments having a H3K4me3 peak.


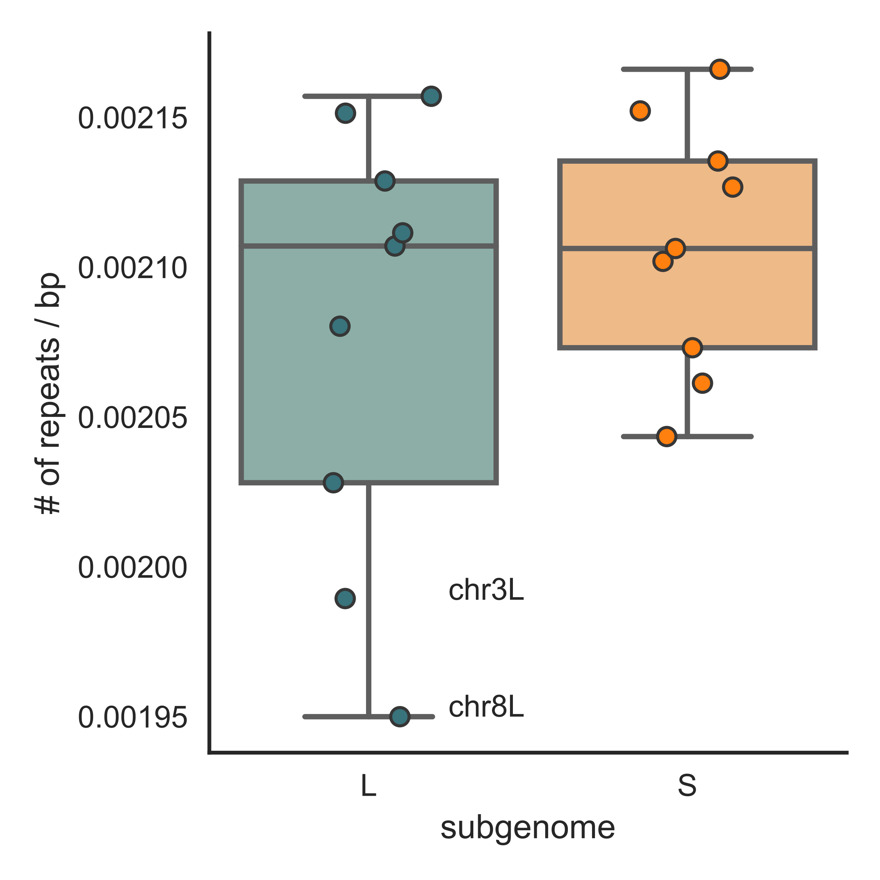


Figure S3. Number of repeats on L and S chromosomes.


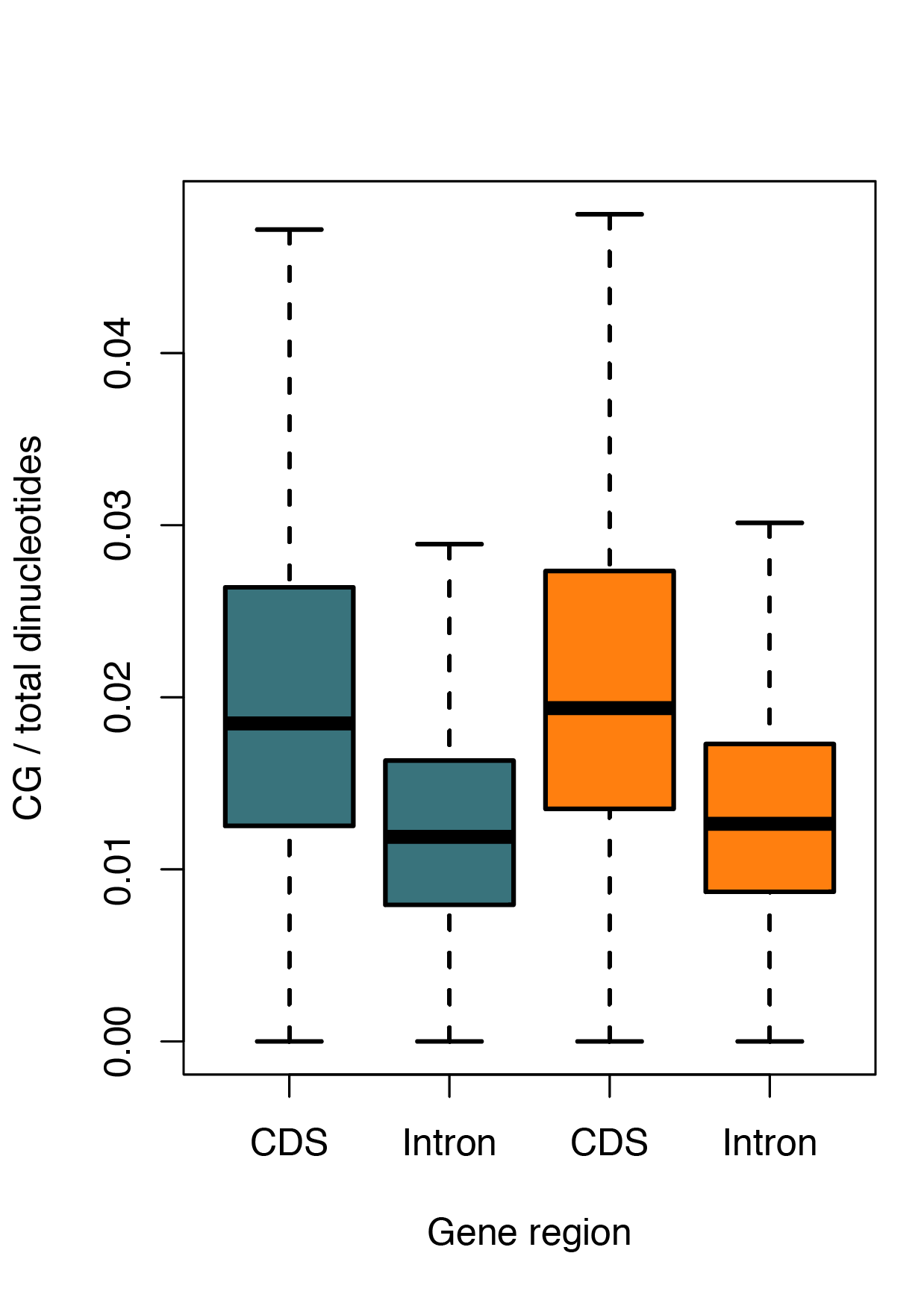


Figure S4: CpG density is in CDS and in introns for both L and S subgenomes


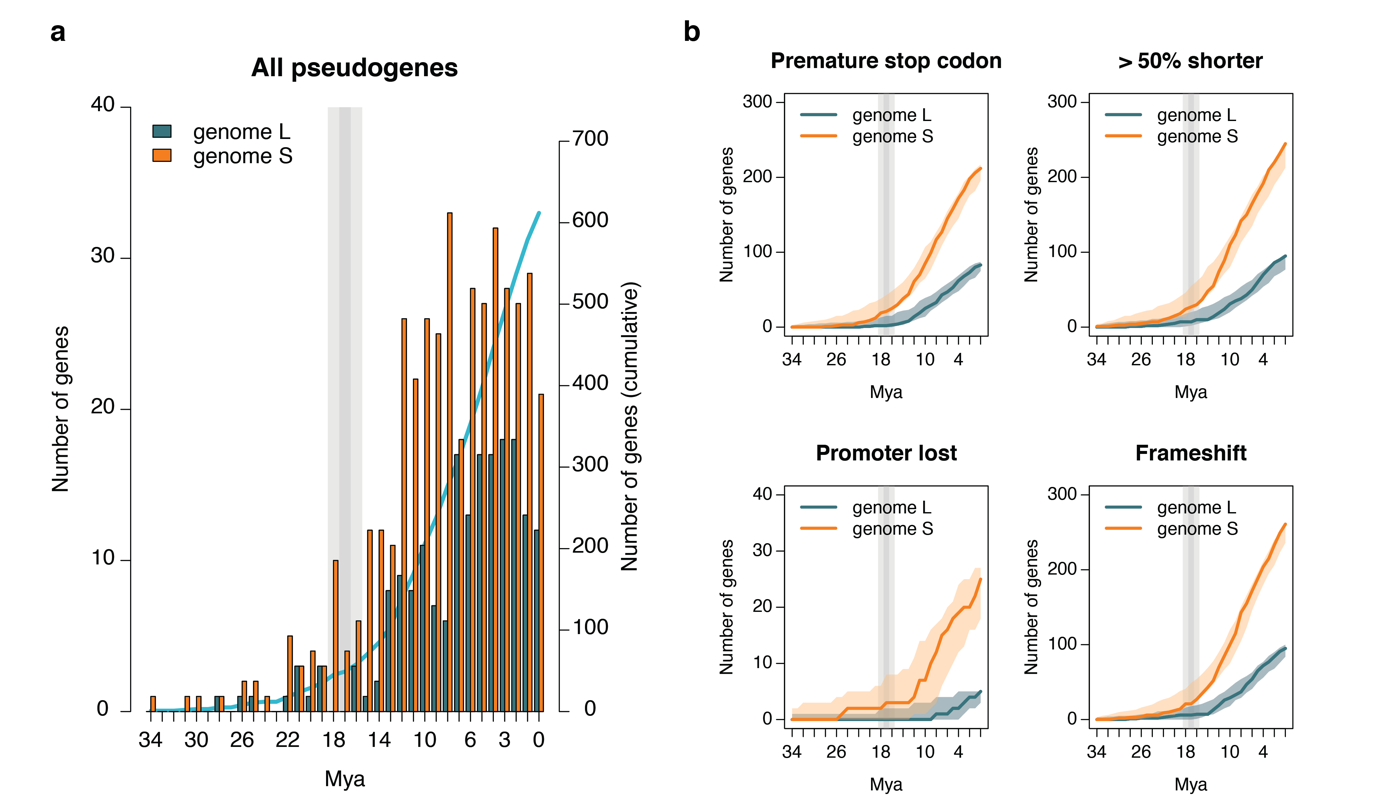


Figure S5: Dating of pseudogenes using extra species. (a) Number of likely pseudogenes (i.e., a gene presenting one or more pseudogene features and 10 times less expression than its homeolog) which have been successfully aligned to their orthologs in human, mouse and chicken, binned by predicted date of pseudogenization event (b) Likely pseudogenes with different (non-exclusive) pseudogene features and their sum over the years. The shaded area depicts the upper and the lower estimates based on the results of the bootstraps.


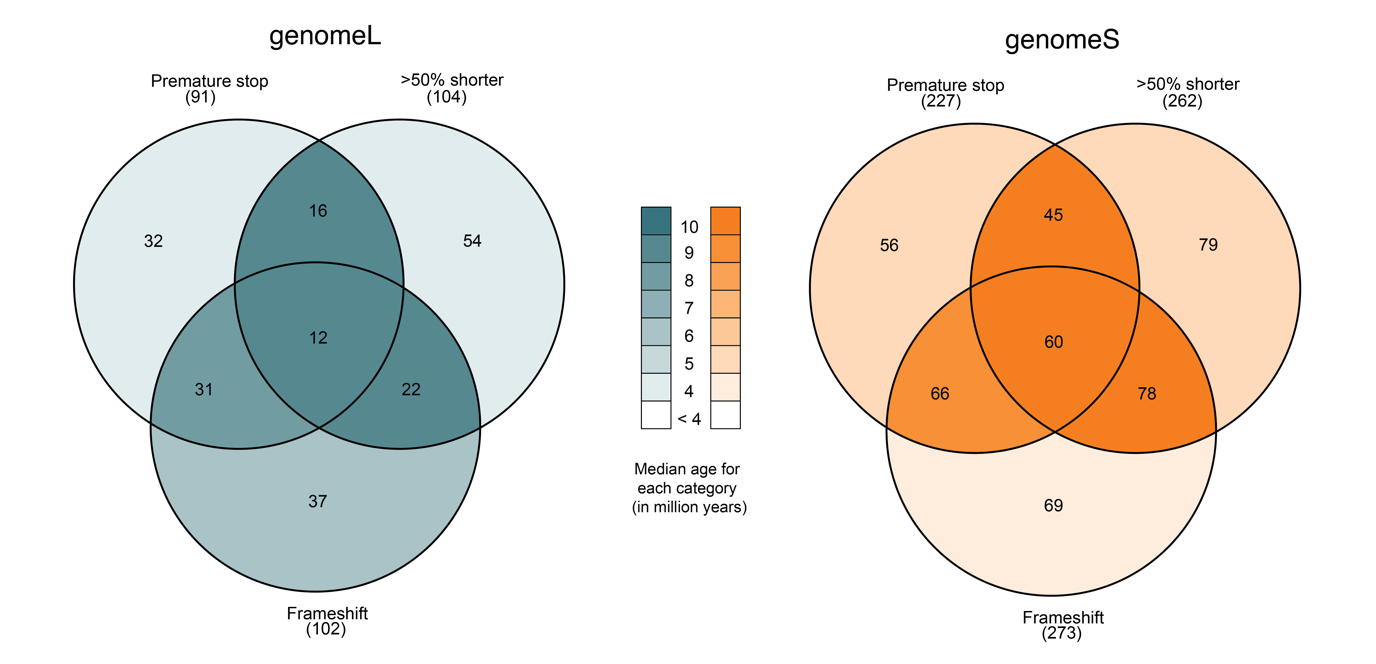


Figure S6: Median age of each category in each subgenome for pseudogenes with one-to-one orthologs in human, mouse and chicken.


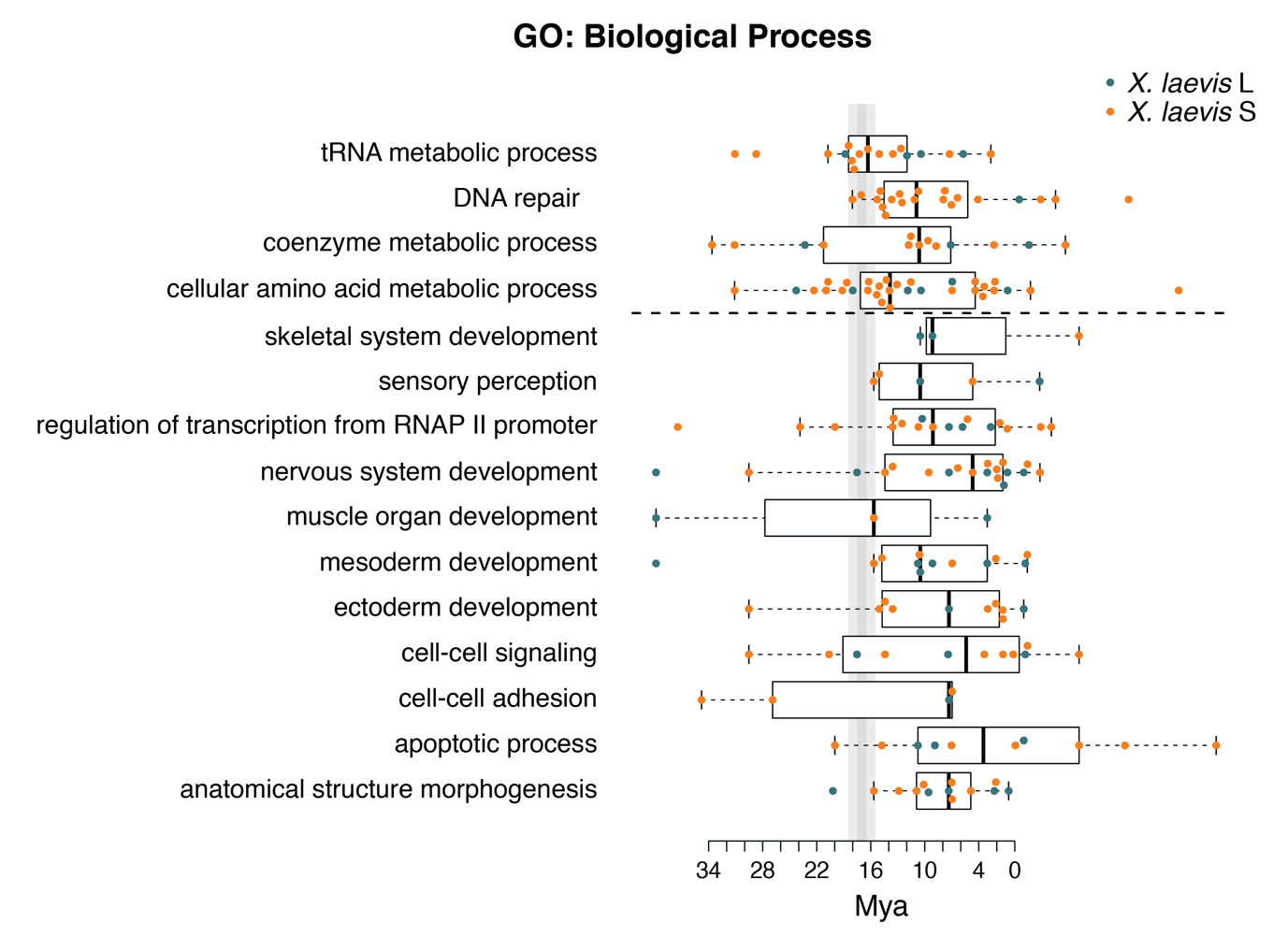


Figure S7: GO term enrichment analysis. Each dot is a pseudogene and its predicted pseudogenization time.


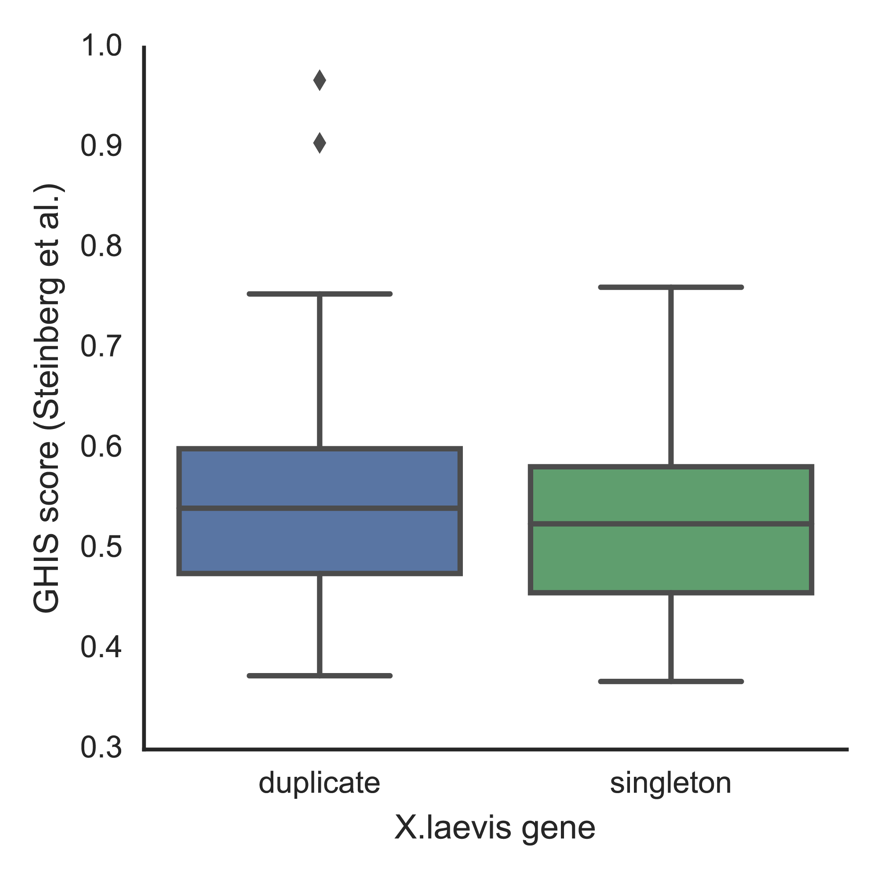


Figure S8: The distribution of genome-wide haploinsufficiency scores (GHIS;

Steinberg et al.) of the human homologs of *X. laevis* genes that are

present in eitehr one copy (singleton) or two (duplicate; homeologs).

Genes that are retained as two copies have a significantly higher GHIS

score (p=1.09e-17, Mann-Whitney U).


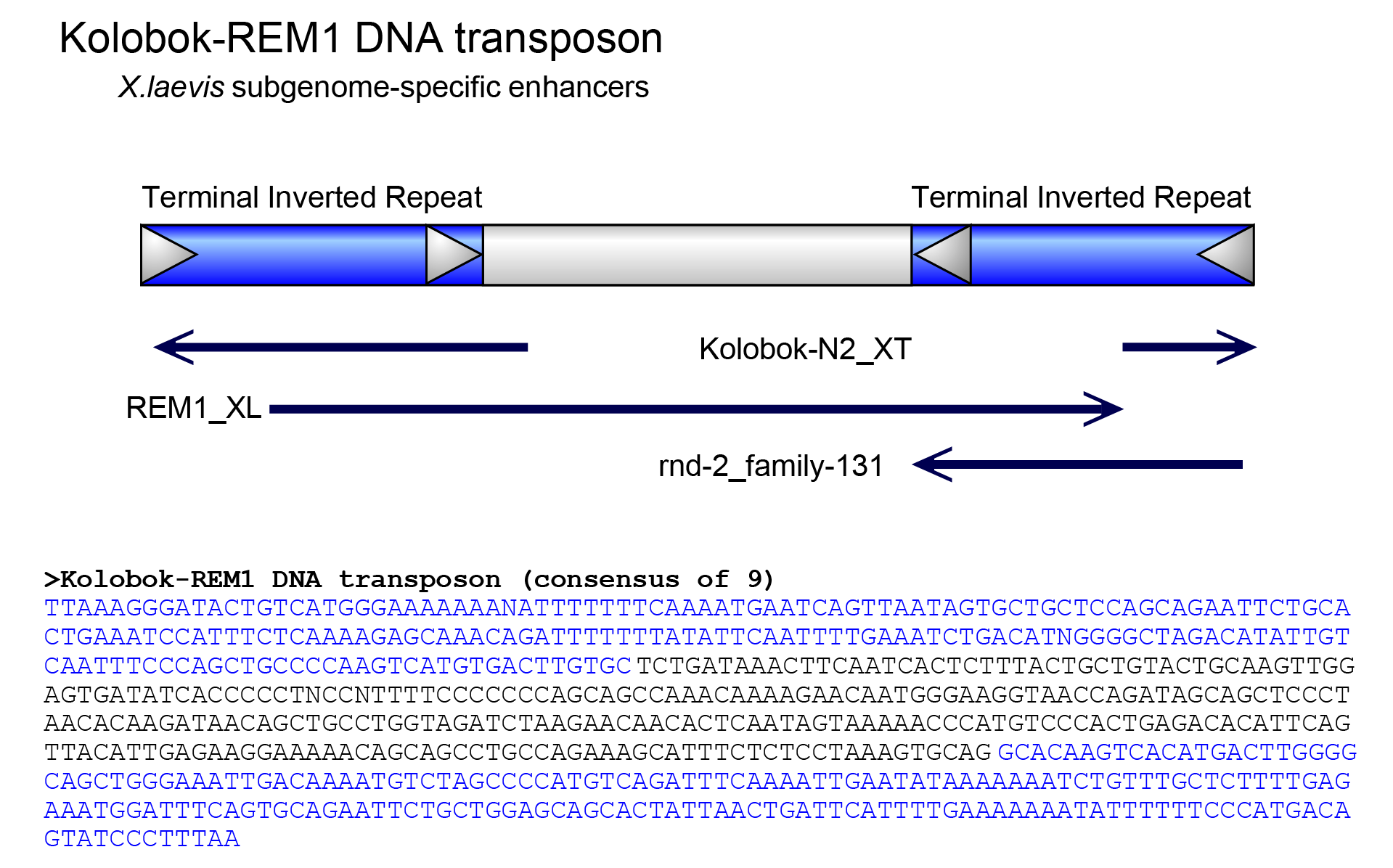


Figure S9. Structure (top) and sequence (bottom) of *X. laevis* Kolobok-REM1 DNA transposon which can recruit the p300 co-activator. The component annotations from the repeat track are shown.


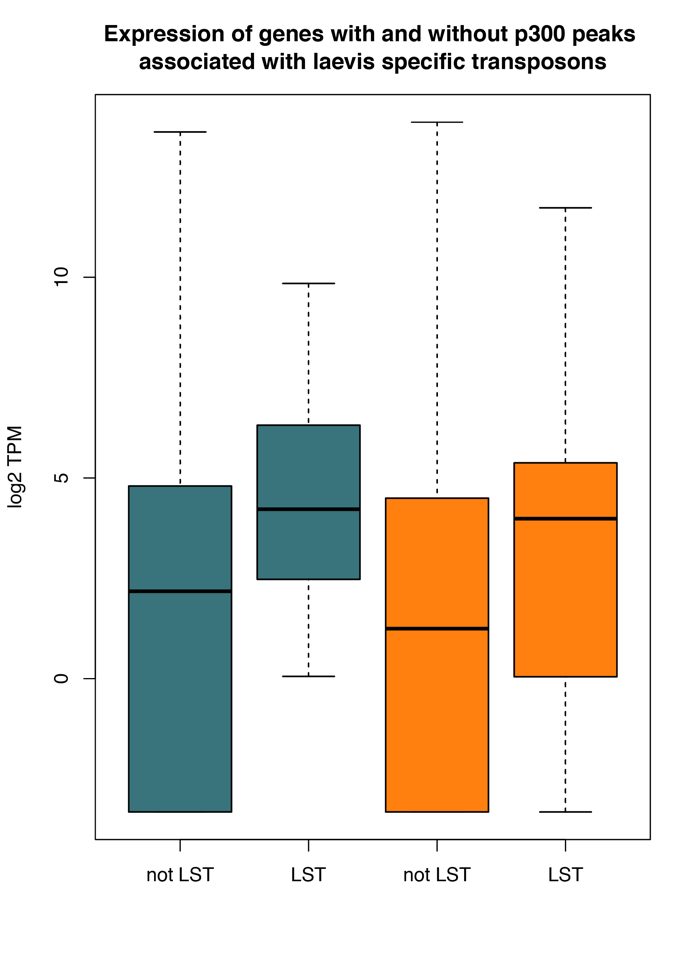


Figure S10: Expression of genes without and with new p300 peaks in laevis specific transposons.


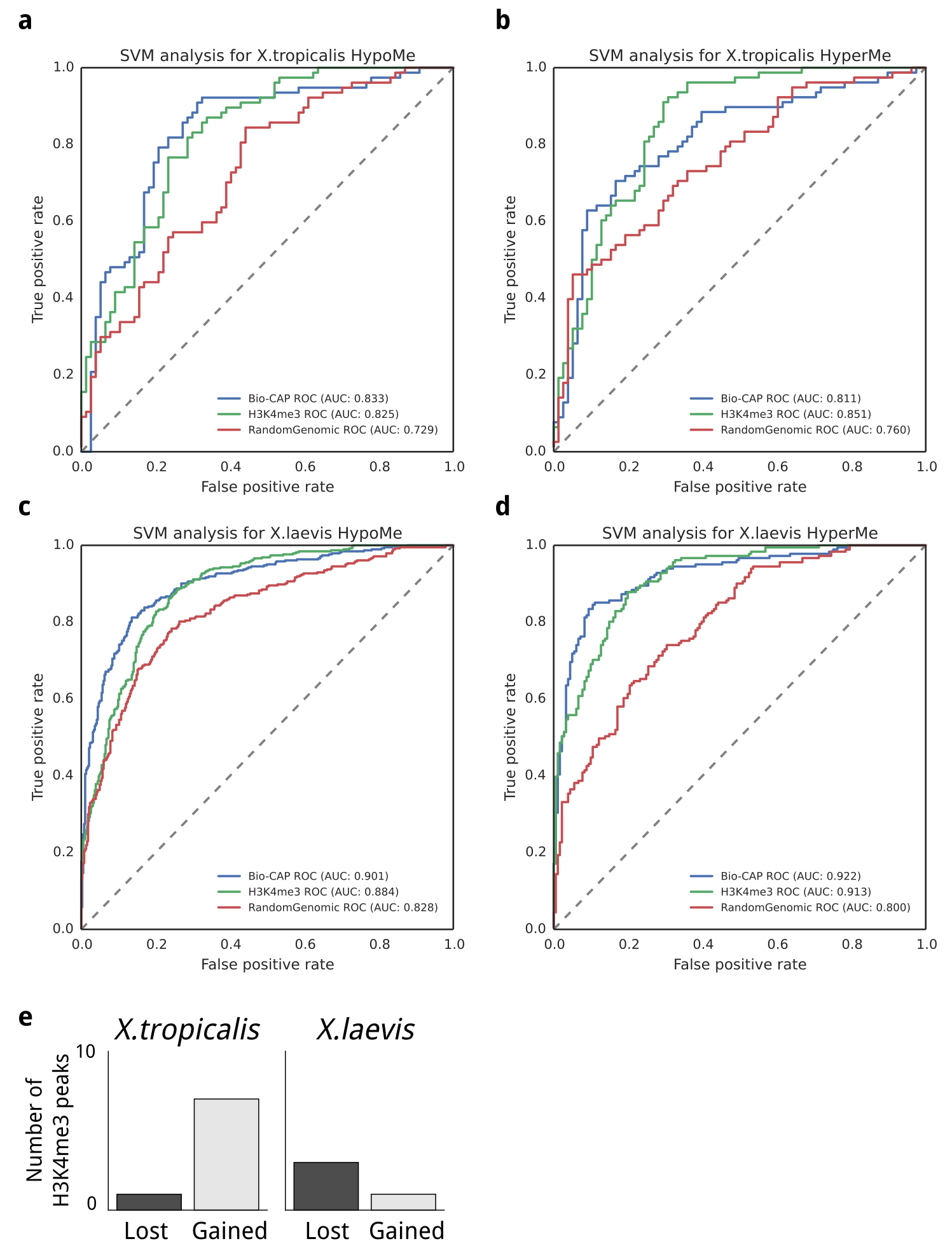


Figure S11. Analysis of differentially methylated regions (DMRs) and H3K4me3 in hybrid embryos. (a-d) Receiver-Operator Curves (ROC) of Support Vector Machines trained on DMR k-mers versus k-mers present in random genomic DNA (red), H3K4me3-positive promoter regions, and unmethylated regions (blue) profiled using Bio-CAP [55]. Areas under the curve (AUC) > 0.5 imply that the SVM distinguishes DMR sequence from other sequences in the case of lost (a) or gained (b) DNA methylation in the *X. tropicalis* subgenome of hybrid embryos, and lost (c) or gained (d) DNA methylation in the *X. laevis* subgenome of hybrid embryos. DMR sequences appear to be different from promoters (H3K4me3 ChIP-seq peaks), unmethylated CpG islands and random genomic sequences, suggesting they represent a specific subset of genomic sequences. However, DMRs with gained DNA methylation (HyperMe) were indistinguishable from DMRs with lost DNA methylation (HypoMe; not shown). A similar result was obtained in the comparison between DMRs present in the *X. laevis* L and S genomes. This indicates that there are no specific sequence signatures distinguishing different types of DMRs (de novo methylated or demethylated, *X. tropicalis* or *X. laevis*). (e) Virtually no gain or loss of H3K4me3 peaks was observed in the subgenomes of LETS hybrid embryos.


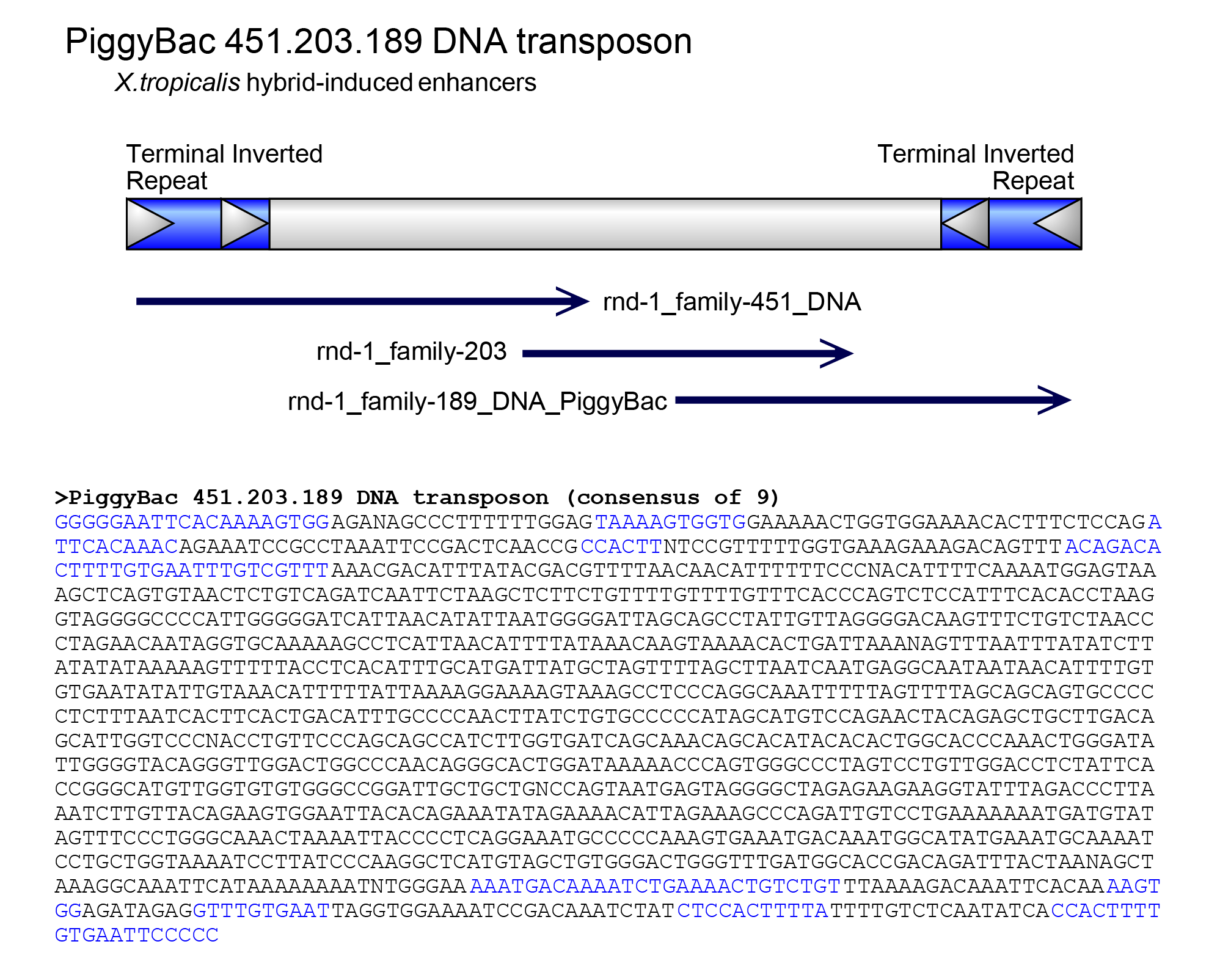


Figure S12. Structure (top) and sequence (bottom) of the *X. tropicalis* PiggyBac 451.203.189 DNA transposon which can recruit the p300 co-activator in LETS hybrid embryos. The component annotations from the repeat track are shown.
