# Supplementary material for: Regulatory remodeling in the allo-tetraploid frog Xenopus laevis
Source: Genome Biol. 2017 Oct 24;18:198. doi: 10.1186/s13059-017-1335-7 (PMC5655803; doi:10.1186/s13059-017-1335-7)
Supplement: Supplementary file 5 — With a supplemental note on the time required to fix pre-existing genomic variation in the population after hybridization. (DOC 38 kb) [file 13059_2017_1335_MOESM5_ESM.doc]

**Supplemental note**

That we find a higher level of SNPs in S than in L is very unlikely to be a relic from the time before the hybridization in which the S species may have had a higher SNP density than L. If we assume an effective population size (Ne) for *X. laevis* of 100,000 and a generation time of 2 years, the expected time to fixation for an initially rare allele would be 4Ne * 2 = 0.8 million years, with a standard deviation of 2.15 Ne [1]. The hybridization event is estimated to have occurred 17 MYA [2] well beyond that expected time and ten standard deviations. Note furthermore that the population went through a bottleneck at the time of the hybridization, and as Ne is the harmonic mean of the (reproducing) population over time, an effective population size of 100,000 is a high estimate. Finally, the 4Ne generations until fixation is for initially rare alleles, while if we assume that, with a bottleneck in the population, the initial frequency of any allele would have been 0.5, the expected time is reduced to fixation to 2.77 Ne [3]. To supplement these analytical considerations, we simulated a Fisher-Wright model of neutral evolution with a population size of 100.000 diploid individuals and initial allele frequencies of 50%. We observed that out of a 1000 cases not a single one remained polymorphic after 1.5 million generations, while the average number of generations required for fixation was as close to the expected value: 277215.

1. Kimura M: **The length of time required for a selectively neutral mutant to reach fixation through random frequency drift in a finite population.** *Genet Res* 1970, **15:**131-133.

2. Session AM, Uno Y, Kwon T, Chapman JA, Toyoda A, Takahashi S, Fukui A, Hikosaka A, Suzuki A, Kondo M, et al: **Genome evolution in the allotetraploid frog Xenopus laevis.** *Nature* 2016, **538:**336-343.

3. Clark AG: **Neutral behavior of shared polymorphism.** *Proc Natl Acad Sci U S A* 1997, **94:**7730-7734.
